# Editorial: Real-world study of multiple sclerosis and related diseases in developing countries

**DOI:** 10.3389/fneur.2023.1336598

**Published:** 2023-11-30

**Authors:** Jinming Han, Edgar Carnero Contentti, Ju Liu

**Affiliations:** ^1^Department of Neurology, Xuanwu Hospital, Capital Medical University, Beijing, China; ^2^Neuroimmunology Unit, Department of Neurosciences, Hospital Alemán, Buenos Aires, Argentina; ^3^Department of Neurology, The First Affiliated Hospital of Zhengzhou University, Zhengzhou, China

**Keywords:** multiple sclerosis (MS), treatment, developing countries, neuromyelitis optica spectrum disorder (NMOSD), MOGAD

Multiple sclerosis (MS) is an inflammatory and neurodegenerative disease of the central nervous system (CNS), serving as the leading cause of non-traumatic neurological disability in young people worldwide. MS and related diseases, such as myelin oligodendrocyte glycoprotein antibody-associated disease (MOGAD) and neuromyelitis optica spectrum disorder (NMOSD), are associated with an important financial burden due to healthcare resource utilization and the cost of long-term treatment strategies. Potential disparities and barriers to health care in developing countries should not be ignored (1), while populations in resource-limited regions are usually understudied. Notably, early diagnosis of these diseases and access to optimal management and long-term therapies are critical to minimizing disability over time in affected patients.

For example, in this Research Topic, Luo et al. established clinical prognostic models to predict visual disability in Chinese patients with NMOSD using retrospective medical data, with serum aquaporin-4 positivity, optic neuritis at disease onset, and older age of onset being significant risk factors. These predictors need to be validated in external disease cohorts, and then neurologists can decide whether these selected patients with NMOSD should be treated with more effective drugs during the early disease course.

Boldrini et al. complemented the scope of this Research Topic by describing a Brazilian patient with NMOSD (presenting short myelitis) who had increased expression of GzmB in circulating T and B cells during rituximab treatment and further developed fatal venous thromboembolism, highlighting the measurement of GzmB expression in circulating lymphocytes as a potential safety biomarker during the treatment of NMOSD.

Duan et al. summarized the rehabilitation assessment and treatment in clinical practice to promote functional recovery in MS patients. Some novel rehabilitation technologies, including transcranial magnetic stimulation, transcranial direct current stimulation, virtual reality, robot-assisted gait training, and telerehabilitation, were discussed in this review. These technologies should be used to improve the quality of life of patients in developing countries.

Pandit et al. compared the live cell-based assay (unavailable in many developing countries) and the fixed cell-based assay for the diagnosis of MOGAD in India, and the results showed a high efficiency of the fixed cell-based assay for the detection of serum MOG-IgG in the 1:10 dilution.

Differences in management and access to the best health care are a significant problem worldwide, including in developing countries. The work included in this Research Topic highlights the importance of collaborative efforts to improve the quality of life of people with MS and related disorders in developing countries.

## Author contributions

JH: Writing—original draft. EC: Writing—review & editing. JL: Writing—review & editing.
